# Genetic Variants in *RASSF1* (rs2073498), *SERPINE1* (rs1799889), and *EFNA1* (rs12904) Are Associated with Susceptibility in Mexican Patients with Colorectal Cancer: Clinical Associations and Their Analysis In Silico

**DOI:** 10.3390/genes16020223

**Published:** 2025-02-15

**Authors:** César de Jesús Tovar-Jácome, Clara Ibet Juárez-Vázquez, Martha Patricia Gallegos-Arreola, José Elías García-Ortiz, María Eugenia Marín-Contreras, Tomás Daniel Pineda-Razo, Ignacio Mariscal-Ramírez, Oscar Durán-Anguiano, Aldo Antonio Alcaraz-Wong, Rubria Alicia González-Sánchez, Marina Lizbeth Mundaca-Rodríguez, Miriam Yadira Godínez-Rodríguez, Marlín Corona-Padilla, Mónica Alejandra Rosales-Reynoso

**Affiliations:** 1 División de Medicina Molecular, Centro de Investigación Biomédica de Occidente (CIBO), Instituto Mexicano del Seguro Social (IMSS), Sierra Mojada 800, Col. Independencia, Guadalajara 44340, Mexico; cesartovjacome@gmail.com (C.d.J.T.-J.); rubria18@gmail.com (R.A.G.-S.); tosti2306@gmail.com (M.L.M.-R.); ya_dirita@hotmail.com (M.Y.G.-R.); marlinmd2202@gmail.com (M.C.-P.); 2 Dirección Académica Aparatos y Sistemas I, Facultad de Medicina, Decanato Ciencias de la Salud, Universidad Autónoma de Guadalajara (UAG), Zapopan 45129, Mexico; clara.juarez@edu.uag.mx; 3 División de Genética, Centro de Investigación Biomédica de Occidente (CIBO), Instituto Mexicano del Seguro Social (IMSS), Sierra Mojada 800, Col. Independencia, Guadalajara 44340, Mexico; marthapatriciagallegos08@gmail.com (M.P.G.-A.); jose.elias.garcia@gmail.com (J.E.G.-O.); 4 Servicio de Gastroenterología, Hospital de Especialidades, Centro Médico Nacional de Occidente, Instituto Mexicano del Seguro Social (IMSS), Guadalajara 44329, Mexico; meugeniamarinc@yahoo.com.mx; 5 Servicio de Oncología Médica, Hospital de Especialidades, Centro Médico Nacional de Occidente, Instituto Mexicano del Seguro Social (IMSS), Guadalajara 44329, Mexico; dan.pinena.razo@gmail.com (T.D.P.-R.); ignacio.mariscal@imss.gob.mx (I.M.-R.); 6 Servicio de Coloproctología, Hospital de Especialidades, Centro Médico Nacional de Occidente, Instituto Mexicano del Seguro Social (IMSS), Guadalajara 44329, Mexico; droscardurananguiano@gmail.com; 7 Servicio de Patología, Hospital de Especialidades, Centro Médico Nacional de Occidente, Instituto Mexicano del Seguro Social (IMSS), Guadalajara 44329, Mexico; draldoalcarazw@hotmail.com

**Keywords:** colorectal cancer, *RASSF1* (rs2073498), *SERPINE1* (rs1799889), *EFNA1* (rs12904), *RAD51* (rs1801320), in silico analysis

## Abstract

**Background/Objectives:** Colorectal cancer (CRC) is the second leading cause of cancer death worldwide. Variants in genes that regulate processes such as apoptosis and angiogenesis play a significant role in CRC. The objective of this study is to investigate the possible association between *RASSF1* (rs2073498), *SERPINE1* (rs1799889), *EFNA1* (rs12904), and *RAD51* (rs1801320) variants and clinicopathological characteristics of Mexican patients with CRC. **Methods**: DNA of peripheral blood samples was obtained from 631 individuals (349 patients and 282 control individuals). The *RASSF1* (rs2073498), *SERPINE1* (rs1799889), *EFNA1* (rs12904), and *RAD51* (rs1801320) variants were genotyped by polymerase chain reaction-restriction fragment length polymorphism (PCR-RFLP). The association was calculated using the odds ratio (OR) test. *p*-values were adjusted by the Bonferroni test (0.0125). In silico analysis programs, including Combined Annotation Dependent Depletion (CADD), Polymorphism Phenotyping-2 (PolyPhen-2), and Gene Expression Profiling Interactive Analysis (GEPIA), were conducted to predict the functional impact of these variants. **Results:** Patients carrying the G/A genotype of the *RASSF1* (rs2073498) variant showed an association with CRC characteristics, including TNM stages and tumor location (OR > 2.5, *p* = 0.001). Regarding the *SERPINE1* (rs1799889) variant, patients carrying the 5G/4G genotype showed an association between TNM stages and tumor location in the rectum (OR > 1.5, *p* ≤ 0.05). Patients with the G/G genotype for the *EFNA1* (rs12904) variant showed an association with TNM stages and rectal tumor location (OR > 2.0, *p* = 0.001). The *RAD51* (rs1801320) variant had no association with colorectal cancer. **Conclusions**: *RASSF1* (rs2073498), *SERPINE1* (rs1799889), and *EFNA1* (rs12904) variants significantly influence colorectal cancer risk.

## 1. Introduction

Colorectal cancer (CRC) is a disease marked by impaired altered cell growth and programmed cell death mechanisms; this type of cancer is the third most common cancer globally and the second most common cause of cancer-related mortality in 2022. In Mexico, CRC ranks third in incidence and is the deadliest cancer, with 8283 deaths in 2022 [1,2].

Environmental factors, genetic predisposition, and dietary habits influence the development of CRC [3,4]. Regarding genetic factors, there is significant heterogeneity in CRC, which is associated with multiple molecular alterations that can affect processes such as angiogenesis and apoptosis [5,6].

In CRC, tumor cell resistance to apoptosis is essential due to the increased expression of anti-apoptotic genes and the underexpression of pro-apoptotic genes, which leads to uncontrolled cell proliferation [7,8]. In parallel, the expression of angiogenic genes results in the release of growth factors and cytokines that can promote cell proliferation and cause cellular matrix changes that facilitate cancer cell invasion and metastasis [7,9].

During the progression of CRC, genes essential for regulating these critical processes are altered. *SERPINE1* (plasminogen activator inhibitor-1) is one of these genes, located on 7q21.3-22, spanning 12, 178 bp. It encodes a glycoprotein (PAI-1) that plays a role in angiogenesis through fibrinolysis. It also regulates plasmin by preventing the extracellular matrix degradation [10,11,12].

The *SERPINE1* is overexpressed in gastric, colorectal, and renal clear cell carcinoma. [13,14]. Its overexpression correlates with advanced tumor stages, metastasis, and decreased survival in CRC [13,14,15].

One of the most important variants of this gene is rs1799889, localized in −675 to the promoter region. This single guanine nucleotide insertion/deletion variant (5G > 4G) is notable for its correlation with PAI-1 overexpression. This variant has demonstrated substantial associations with other cancers, including endometrial and hepatocellular carcinoma. However, the relationship between this variant and CRC remains unclear [12,16].

Another crucial gene is *EFNA1*, located on 1q22. It is 7024 bp. This gene encodes a transmembrane protein (Ephrin-A1) that interacts with EPH receptors (EphA2) and plays a significant role in angiogenesis [17,18]. This interaction facilitates the formation of new blood vessels around the tumor [17,18,19]. *EFNA1* has been associated with various cancers, and it has been observed to be overexpressed in gastric, colorectal, hepatocellular, renal, cervical, ovarian, esophageal, and laryngeal cancer; this provides further evidence of its significance in the process of oncogenesis [20,21]. The overexpression of *EFNA1* has been related to the early stages of the development of smaller tumors [18,19,20,21,22]. Among the numerous *EFNA1* variants, the rs12904 (A>G) variant situated within the 3′ UTR has been the focus of extensive investigation due to its influence on *EFNA1* expression and tumor angiogenesis [17,18,22]. Nevertheless, the evidence supporting an elevated risk of gastric cancer and CRC associated with this variant remains inconclusive [20,22,23].

The *RASSF1* gene (RAS-binding domain family protein 1) is located on 3p21.3, spanning 11,199 bp. It encodes a protein (RASSF1) that has seven isoforms. The RASSF1A isoform is the most extensively studied and is involved in apoptosis due to DNA damage [24,25,26,27,28,29,30,31]. *RASSF1* underexpression has been observed in several cancer types, including lung, breast, bladder, prostate, and CRC [32,33]. This underexpression is related to an advanced tumor stage, poor tumor differentiation, and worse survival in CRC [33,34].

One of the most extensively studied variants in the *RASSF1* gene is rs2073498 (G>T). This variant significantly influences the regulation of RASSF1, resulting in a nonsense alteration in exon 3 that alters the alanine at position 133 to serine. This variant is associated with an elevated risk of hepatocellular, esophageal, gastric, breast, and lung cancer [24,31,33,35]. The role of this factor in CRC has yet to be fully elucidated, and its correlation with the disease has not been demonstrated to be statistically significant. Therefore, further investigation into other populations is essential to gain insight into its potential involvement in this cancer [28,29,31,35,36].

The *RAD51* gene, located at 15q15.1, is 30 kilobases long. This gene plays a crucial role in homologous recombination and DNA repair, ensuring genome integrity and preventing apoptosis [37,38,39,40,41,42,43,44,45]. Its overexpression has been linked to an increased risk of developing certain types of cancer, including breast and lung cancer [46]. Additionally, studies have shown that elevated levels of *RAD51* are associated with a poorer prognosis and reduced responsiveness to treatment [37,39,46,47,48]. This overexpression is promoted by the transcription factor MYC, which is bound in the 5′ untranslated region (UTR) [49].

Consequently, the rs1801320 G>C variant in this region is one of the most extensively investigated variants and is linked to multiple types of cancer. Despite extensive research on its association with CRC, the results remain inconclusive, making it a significant area of focus for CRC research [37,38,40,41,42,45].

Therefore, this study aimed to explore for the first time the possible association between the *RASSF1* (rs2073498), *SERPINE1* (rs1799889), *EFNA1* (rs12904), and *RAD51* (rs1801320) variants and the clinicopathological characteristics of Mexican patients with CRC.

## 2. Materials and Methods

### 2.1. Subjects

The study included 349 clinically diagnosed and histologically confirmed patients with sporadic colorectal adenocarcinoma between 2019 and 2023, according to the Clinical Practice Guidelines in Colon and Rectal Cancer and the clinicopathological criteria of Hospital de Especialidades Médicas del Centro Medico Nacional de Occidente del Instituto Mexicano del Seguro Social (IMSS) in Guadalajara, Mexico. Pathological staging and grading of the tumors were carried out by the tumor-node-metastasis (TNM) classification. The control group included 282 healthy, unrelated individuals age-matched with the patients’ group and with a confirmed negative colonoscopy for malignancy. All samples were collected consecutively from individuals treated at this center between 2018 and 2022. None of the included patients had undergone chemotherapy or radiotherapy during sampling. Exclusion criteria for patients and controls included a negative diagnosis of autoimmune or inflammatory bowel disease and a family history of any known hereditary cancer syndrome.

The Comité local de Investigación en Salud 1305 at Instituto Mexicano del Seguro Social (IMSS) approved the study. It was approved with the registration number of the project (R-2018-1305-001). The study was conducted according to national and international ethical standards. All the participants signed the informed consent for participation in this study. A standard epidemiological questionnaire allowed us to collect personal data, including age, sex, tobacco and alcohol consumption, and familial history. Information on the clinical and pathological characteristics of the patients was obtained from hospital records.

### 2.2. Genotyping

Genomic DNA was isolated from peripheral blood using standard methods [50]. The variants *RASSF1* rs2073498 (Ala133Ser), *SERPINE1* rs1799889 (5G/4G), *EFNA1* rs12904 (A>G), and *RAD51* rs1801320 (G>C) were genotyped by polymerase chain reaction-restriction fragment length polymorphism (PCR-RFLP), using the following primer pairs: For the *RASSF1* gene, rs2073498-F: 5′-GTACATCAGGGACAGGGGGC-3′ and rs2073498-R: 5′-CATGAAGAGGTTGCTGTTGATC-3′ [51]; for the *SERPINE1* gene, variant rs1799889-F: 5′- CACAGAGAGAGTCTGGCCACGT-3′ and rs1799889-R: 5′- CCAACAGAGAGGACTCTTGGTCT-3′ [52]; for the *EFNA1* gene, variant rs12904-F: 5′-ACAGGCTGAAGAGAGGGACA-3′ and rs12904-R: 5′-AACTTCTCTGTGGCAGCTCC-3′ [53]; and for the *RAD51* gene, rs1801320-F: GGAACTGCAACTCATCTGGG-3′ and rs1801320-R: 5′-TCACACACTCACCTCGGTC-3′ [54].

The PCR reaction for *RASSF1* rs2073498, *SERPINE1* rs1799889, *EFNA1* rs12904, and *RAD51* rs1801320 variants was performed for 35 cycles in a 10 μL volume containing 100 ng DNA, 10X buffer (500 mM KCl, 100 mM Tris-HCl, and 0.1% TritonTM X-100), 2.0 mM MgCl2, 200 mM dNTPs, 1 pM of each primer, and 2U Taq DNA Polymerase (Thermo, Waltham, MA, USA, et al.,) according to the following amplification program: initial denaturation at 94 °C for 5 min, 35 cycles of denaturation at 94 °C for 30 s, annealing at 56 °C for (rs2073498), 60 °C for (rs1799889), 62 °C for (rs12904), and 58 °C for (rs1801320) respectively for 45 s, and extension at 72 °C for 30 s followed by final extension at 72 °C for 10 min. Five microliters of each PCR product were digested using 5U of the following restriction enzymes: rs2073498 (*AluI*), rs1799889 (*BslI*), rs12904 (*SspI*), and rs1801320 (*BstNI*) (New England Biolabs, Ipswich, MA, USA) according to the manufacturer’s instructions and finally separated in 6% polyacrylamide gels. Genotypes were identified according to the descriptions by [51,52,53,54]. To ensure the quality of the genotyping processes, approximately 10% of the random samples were reprocessed, and the results were found to be 100% consistent.

### 2.3. Statistical Analysis

Genotype and allele frequencies were estimated by direct counting in both groups. The Chi-square test assessed the Hardy–Weinberg equilibrium (HWE) and differences in genotype and allele distributions with the clinical features of patients and controls. Statistical analysis included an odds ratio test. Associations of genotypes or alleles with CRC, stratified with demographic and clinicopathological characteristics, were calculated by odds ratio (OR) and confidence intervals (CI) in an SPSS v25.0 software package (SPSS Inc., Chicago, IL, USA). For all statistical analyses, *p* < 0.05 was considered significant. A Bonferroni correction test was applied to adjust the *p*-values (*p* < 0.0125).

### 2.4. In Silico Analysis of Variants Analyzed

To assess the impact of genetic variants, we used the Combined Annotation Dependent Depletion (CADD) (v1.7), an in silico tool, which evaluates potential deleteriousness by analyzing their positions in the human genome (GRCh38). CADD (v1.7) provides raw and c-phred scores to estimate the likelihood of a variant being deleterious, with higher c-phred scores indicating a higher probability of being deleterious. CADD (v1.7) integrates more than 60 annotations to classify variants, providing a comprehensive approach. Some of the most important are SIFT (v5.2.2), PolyPhen-2 (v2), ESMscore (v1), and mirSVR (v1.2). We also accessed the PolymiRTS (v3.0) database for miRNA-related information and used GEPIA(v2) and GTEx (v10) for gene expression analysis across different tissues and stages.

The PolymiRTS database was accessed to complement the information and identify miRNAs related to the UTR sections of the variants analyzed. GEPIA (v2) was used to analyze gene expression levels by stage, and GTEx (v10) was used to analyze expression in different tissues.

## 3. Results

### 3.1. Clinicopathological Characteristics of the Subjects Included in the Study

Table 1 shows the clinicopathological characteristics of study subjects. The mean age observed was 58.34 (±12.32) for the CRC group and 59.76 (±13.28) years in the control group (*p* = 0.165). Sex did not show significant differences between the groups analyzed (*p* > 0.05). However, tobacco and alcohol consumption showed significant differences between these groups (*p* = 0.001).

### 3.2. Genotype Frequencies Analysis of the RASSF1, SERPINE1, EFNA1, and RAD51 Variants and In Silico Analysis

Table 2 compares the *RASSF1*, *SERPINE1*, *EFNA1*, and *RAD51* variants analyzed in the genomic DNA of patients with CRC and controls. The four variants studied in the control group were in Hardy–Weinberg equilibrium. For the rs2073498 (Ala133Ser) variant in the *RASSF1* gene, we observed that the patient carriers of Ala/Ser and Ser/Ser genotypes showed an increased susceptibility for developing CRC (OR = 2.58; 95% CI = 1.85–3.60, *p* = 0.001 and OR = 8.84; 95% CI = 3.62–21.56, *p* = 0.001), respectively, and this association was also evident under the dominant model of inheritance, Ala/Ser + Ser/Ser vs. Ala/Ala (OR = 2.93; 95% CI = 2.12–4.06, *p* = 0.001). Allelic frequencies were also significantly different, demonstrating that carriers of the Ser allele have increased susceptibility for developing CRC (OR = 2.41; 95% CI = 1.86–3.11, *p* = 0.001).

Regarding the association of clinicopathologic features with this variant, a statistical significance was observed in individuals over 50 years and carrying the Ala/Ser and Ser/Ser genotypes (OR = 2.67, 95% CI = 1.86–3.84, *p* = 0.001 and OR = 11.36, 95% CI = 4.31–29.96, *p* = 0.001), respectively (Table 3). Males and females with the Ala/Ser genotype have a significantly increased susceptibility to developing CRC (*p* = 0.001). CRC patients with tobacco consumption and with the Ala/Ser genotype have significantly increased susceptibility to developing CRC (OR = 6.56, 95% CI = 2.84–15.15, *p* = 0.001). The analysis of disease development reveals that individuals carrying the Ala/Ser and Ser/Ser genotypes have a significantly increased susceptibility to developing CRC in early and advanced TNM stages (*p* = 0.001). Patient carriers of the Ser/Ser genotype and colon tumor location showed an increased susceptibility to developing CRC.

In silico analysis of the *RASSF1* variant (rs2073498) by the CADD tool demonstrates a cumulative score of 2.85 and a c-phred score of 20.6; within the annotations that CADD evaluated for this variant are the SIFT score (0.26) and PolyPhen-2 (0.013, 2). An EsmScoreMissense (−5.569) (Supplementary Table S1).

An analysis of *RASSF1* expression profiles in colorectal adenocarcinoma (COAD) and rectal adenocarcinoma (READ) by the GEPIA web-based tool (Supplementary Figure S1a) revealed modest underexpression in patients, though this was not statistically significant. Subsequently, expression differences across tumor stages were analyzed using GEPIA (Supplementary Figure S2a), with an F value of 2.72 and a *p*-value of 0.044. Finally, a gene expression quantitative trait loci (eQTL) analysis for the *RASSF1* rs207349 variant on 660 individuals from the GTEx repository (Supplementary Figure S3a) in whole blood (*p* = 0.36), transverse colon (*p* = 0.093), and sigmoid colon (*p* = 0.076).

Regarding the *SERPINE1* (rs1799889) variant, patients with the 5G/4G genotype showed increased susceptibility to developing CRC (OR = 1.78; 95% CI = 1.23–2.56, *p* = 0.002). This association was also evident under the dominant model of inheritance (5G/4G + 4G vs. 5G/5G) (OR = 1.76; 95% CI = 1.24–2.50, *p* = 0.001) (Table 2).

Concerning the association of clinicopathologic features with this variant, a statistical significance was observed in male individuals carrying the 5G/4G genotype (OR = 2.34, 95% CI = 1.42–3.85, *p* = 0.001). Alcohol consumption shows statistical significance (OR = 4.16, 95% CI = 1.74–9.89, *p* = 0.001). The analysis of disease development reveals that individuals carrying the 5G/4G genotype are marginally susceptible to developing CRC in advanced TNM stage (IV) (OR = 2.19, 95% CI = 1.26–3.81, *p* = 0.006), respectively. The analysis based on tumor location indicates that individuals with the 5G/4G genotype are more susceptible to developing tumors in the rectum (OR = 2.00; 95% CI = 1.35–2.97, *p* = 0.001) (Table 4). The *SERPINE1* rs1799889 variant was evaluated using the CADD tool (Supplementary Table S1), and it exhibited a raw score of 1.96 and a c-phred score of 16.61. The CADD’s annotation GerpRS shows (346.19) and *p*-value < 0.05.

*SERPINE1* expression was analyzed in patient and control samples using GEPIA (Supplementary Figure S1b), which revealed a slight overexpression in the COAD and READ groups (*p* > 0.05). Examining tumor stages with GEPIA (Supplementary Figure S2b) shows an F value of 5.28 and a *p* = 0.001.

An eQTL analysis of whole blood, transverse colon, and sigmoid colon using 660 GTEx samples regarding the *SERPINE1* rs207349 variant revealed no related expression data.

Concerning the *EFNA1* (rs12904) variant, we observed that the patient carriers of the G/G genotype showed an increased susceptibility for developing CRC (OR = 2.23; 95% CI = 1.44–3.46, *p* = 0.005, and this association was also evident under the dominant model of inheritance (A/G + G/G vs. A/A) (OR = 1.68; 95% CI = 1.17–2.41, *p* = 0.005). Allelic frequencies were also significantly different, demonstrating that carriers of the G allele have increased susceptibility for developing CRC (OR = 1.52; 95% CI = 1.22–1.91, *p* = 0.001) (Table 2). Statistical significance was observed in female individuals carrying the G/G genotype (OR = 2.76, 95% CI = 1.40–5.44, *p* = 0.004) regarding the association of clinicopathologic features with this variant. The analysis of disease development reveals that individuals carrying the G/G genotype have an increased susceptibility to developing CRC in the advanced TNM stage (IV) (OR = 3.04, 95% CI = 1.60–5.76, *p* = 0.001), respectively. The analysis based on tumor location indicates that individuals with the G/G genotype are more susceptible to developing tumors in the rectum (OR = 2.35; 95% CI = 1.47–3.74, *p* = 0.001) (Table 5).

The *EFNA1* rs12904 variant was analyzed using the CADD in silico tool (Supplementary Table S1), which showed a raw score of 2.33 and a c-phred score of 18.26. CADD annotations were analyzed in this variant (target score = 9), (mirSVR score = −1.088).

Further exploration via the PolymiRTS database (Supplementary Table S2) identified eight conserved miRNAs linked to this variant, classified as disruptive. Their context scores were miR-200b = −0.17, miR-200c = 0.17, miR-429 = −0.17, miR-4750 = −0.246, miR-8084= −0.002, and miR-888 = −0.14, miR-374 = 0.028, and miR-655 = 0.28, and the main target genes of these miRNAs.

The expression of *EFNA1* was examined in COAD and READ tissue by GEPIA (Supplementary Figure S1c). Overexpression in patients was demonstrated, though not statistically significant. A posterior analysis of expression by tumor stages in GEPIA (Supplementary Figure S2c) revealed an F value of 3.67, *p* = 0.012. A whole blood eQTL assay for the *EFNA1* rs12904 variant in whole blood (*p* = 0.987), transverse colon (0.0783), and colon sigmoid (*p* = 0.001) (Supplementary Figure S3b).

Regarding the *RAD51* (rs1801320) variant, no statistical association was observed for any genotype or allele (Table 2), and no significant differences were observed when comparing sex, age, alcohol, tobacco consumption, TNM stage, and tumor location among groups.

Table 6 shows the results of multiple logistic regression analysis, including confounding characteristics. Age, tobacco, and alcohol consumption were statistically significant in the presence of the three variants associated with *RASSF1* (rs2073498), *SERPINE1* (rs1799889), and *EFNA1* (rs12904), suggesting that these variables increase the risk and susceptibility of developing CRC; however, when the dominant models of the variants were analyzed in the presence of these confounding variables, it was found that the dominant models of *EFNA1* and *RAD51* were not statistically significant.

## 4. Discussion

This study analyzed the clinicopathological characteristics of patients and the control group. Statistically significant differences were observed for age, smoking, and alcohol consumption. These findings related to the significant differences observed between age groups are consistent with those reported by [55], who observed substantial differences in tobacco consumption between colorectal cancer patients and controls. Moreover, other studies have shown significant associations among age, tobacco, alcohol consumption, and colorectal cancer [56,57]. These results are consistent with international clinical practice guidelines, highlighting age, tobacco, and alcohol as significant risk factors for CRC [58,59]. In addition, this result highlights the considerable impact of age, smoking, and alcohol consumption on colorectal cancer risk, emphasizing the necessity for targeted prevention strategies. Recently, the variants *RASSF1* rs2073498, *SERPINE* rs1799889, *EFNA1* rs12904, and *RAD51* rs1801320 have been analyzed in cancer, getting variable results in multiple ethnicities [11,16,20,22,34,39,44,45,53,54,60] Therefore, in this study, we examined for the first time in the Mexican population the genotype distribution of four variants, rs2073498, rs1799889, rs12904, and rs1801320, in the *RASSF1, SERPINE, EFNA1*, and *RAD51* genes. The results show that the *RASSF1*, *SERPINE*, and *EFNA1* variants are associated with CRC’s development and clinicopathological characteristics.

Regarding the *RASSF1* rs2073498 (Ala133Ser) variant, we observed that patient carriers of Ala/Ser and Ser/Ser genotypes are more susceptible to developing CRC. Previous studies have associated this variant with an increased risk of hepatocellular, esophageal, gastric, breast, and lung cancer [25,28,61]. Our study found an increased susceptibility to CRC in individuals with Ala/Ser and Ser/Ser genotypes. The biological function may be linked to an alteration in the ATM region, which is crucial for its activation and tumor suppressor activity. The 133Ser allele has an altered secondary structure that prevents ATM activation and subsequent phosphorylation after DNA damage. This reduced phosphorylation in ATM affects the regulation of cytokinesis and microtubule stability, leading to decreased stability of p73 and p53, reflected in lower expression of the target gene p21. This may result from reduced activation of MST1/2, impacting p53 deacetylation [31,62,63,64].

In silico analysis using the CADD program indicates a high potential deleterious effect. The principal annotations analyzed in CADD with this variant show that PolyPhen-2 classifies this variant as benign, and SIFT scores it as a tolerated amino acid change. However, the EsmScoreMissense score suggests a deleterious effect due to factors such as the hydrophobicity, charge, and size of amino acids. Regarding gene expression levels, the initial GEPIA web-based program analysis revealed a tendency towards underexpression in COAD and READ tissues. However, this variation was not statistically significant. However, the stage analysis conducted with GEPIA showed a significant difference in expression between tumor stages. Additionally, the eQTL analysis from the GTEx repository found no significant differences in expression levels for the *RASSF1* rs207349 variant in whole blood, transverse colon, or sigmoid colon tissues.

According to bioinformatics analysis, the missense variant does not affect the protein structurally. However, it does show that there is an effect at the amino acid level in charge size and hydrophobicity; the studies show that the change in the protein could be at the secondary structure level and that this modifies the hydrophobicity and possibly the charge of the protein, making it not transmit the signal to the ATM pathway, which is in agreement with our result. On the other hand, the in silico analysis showed no significant difference in expression in colon or rectal tissue. However, there is a trend towards underexpression, consistent with the literature showing that this gene is progressively underexpressed, consistent with the significant difference in expression levels by tumor stage. Our findings underscore the importance of the rs2073498 variant of *RASSF1*, potentially due to alterations in the protein’s secondary structure. This underscores the importance of combining genetic, bioinformatics, and gene expression analyses to gain a deeper understanding of the mechanisms of the variants and their contribution to CRC.

The rs1799889 (5G/4G) variant of the *SERPINE1* gene is associated with the overexpression of PAI1, causing alterations that play an essential role in various cellular functions, mainly fibrinolysis and angiogenesis [12,16,65]. In this study, we found that patients with the presence of the 5G/4G genotype of this variant have an increased susceptibility to developing CRC, which is different from what is reported in most studies where an association between this variant and the development of colorectal cancer has not been identified [10,65,66,67,68,69]. The biological implications of the promoter variant with the 4G allele are an increase in *SERPINE1* expression, while the 5G allele has the opposite effect, reducing expression. The combination of the 5G and 4G alleles may be more dynamic, explaining these results findings [10,65,66,67,68,69].

The CADD in silico analysis revealed that the rs1799889 variant of the *SERPINE1* gene carries a moderate risk of deleterious impact (rawScore = 1.96) (c-phred = 16.61) and is evolutionarily conserved (GerpRs = 346.19). The expression analyses by GEPIA indicated a trend towards overexpression of *SERPINE1* in COAD and READ tissues. However, the stage analysis conducted with GEPIA showed a significant difference in expression between tumor stages. Additionally, the eQTL analysis from the GTEx repository found no significant differences in expression levels for the *SERPINE1* rs1799889 variant in whole blood, transverse colon, or sigmoid colon tissues.

The in silico analysis revealed an overexpression in colon and rectal tissue, which is consistent with the finding that the change in the promoter is related to increased overexpression in colorectal cancer. The in silico analysis showed that the risk of deleteriousness is low, which may be associated with the different functional behaviors in various diseases. This may be due to a complex relationship between the variant and environmental or clinical factors, consistent with a moderate deleterious effect. The results of our study highlight the necessity for further investigation to elucidate the function of the rs1799889 variant in CRC. It is crucial to consider the role of gene variants that regulate gene expression as potential biomarkers. Additionally, the complexity of multifactorial interactions underscores the significance of precision medicine.

Regarding the *EFNA1* (rs12904) variant, increased susceptibility of CRC patients was observed in the presence of a G/G genotype, which contrasts with the studies [20,55], which found no significant association of this variant with CRC.

According to the CADD analysis, the *EFNA1* (rs12904) variant has a moderate impact on the regulation of the expression of this gene (rawscore = 2.33) (c-phred = 18.26). According to the CADD annotations (Target = 9), (MirSVRscore = −1.08), this impact is through its regulation by miRNAs since it is known that this change can modify the binding site of *EFNA1* with miR-200c. This change is related to a higher expression of *EFNA1*, which favors an increase in angiogenesis and the development of CRC [20,55]. These observed effects are consistent with the PolymiRTS in silico analyses, which identified eight microRNAs associated with this variant. The following miRNAs were identified: miR-200b, miR-200c, miR-374, miR-429, miR-4750, miR-655, miR-8084, and miR-888. This finding extends previous observations by [55], which reported an association with only miR-200c and miR-429. The identification of six additional miRNAs suggests that the rs12904 variant may exert broader control in the post-transcriptional regulation of *EFNA1*, which in turn is reflected in its considerable deleterious effect score.

These miRNAs play a pivotal role in the pathogenesis of cancer. The miR-200 family, particularly miR-200c and miR-200a-3p, has been demonstrated to regulate the ephrin genes *EFNA1* and *EFNA5*, thereby influencing tumor growth and metastasis [70]. Other significant miRNAs include miR-374c-5p, which promotes cell proliferation, and miR-429, which affects angiogenesis [71,72]. MiR-4750-3p regulates apoptosis, influencing cancer cell survival, while miR-655-3p is involved in cancer-related inflammatory responses [73,74]. Although miR-8084 needs more study, it may affect gene regulation in certain cancers [75]. Lastly, miR-888-3p contributes to cell signaling and development [76]. Exploring these miRNAs could lead to new insights for cancer biomarkers and therapies.

Conversely, the gene expression levels of the initial analysis using the GEPIA web-based program revealed a tendency towards overexpression in COAD and READ tissues, but this variation was not statistically significant. However, the stage analysis conducted with GEPIA showed a significant difference in expression between tumor stages. Additionally, the eQTL analysis from the GTEx repository found no significant differences in expression levels for the *EFNA1* rs12904 variant in whole blood, transverse colon, or sigmoid colon tissues.

Finally, the rs1801320 variant of the *RAD51* gene was not found to have a statistically significant association with the development of CRC. This agrees with the findings of Mucha et al. [45], who also found no significant association between this variant and CRC. However, it differs from Krupa et al. [44], who found a statistically significant association between the C/C genotype and a lower susceptibility to CRC. This could be due to differences between the populations analyzed.

When the confounding variables, such as age, tobacco, and alcohol consumption, were analyzed, statistical significance was observed in patients with CRC. This is related to the international CRC treatment guidelines, where tobacco and alcohol consumption is considered one of the main risk factors [58,59].

This study identifies specific genetic variants (*RASSF1* rs2073498, *SERPINE1* rs1799889, and *EFNA1* rs12904) associated with colorectal cancer in the Mexican population and highlights their value as potential biomarkers for late-stage prognosis. Integrating these findings into the field of personalized medicine could markedly enhance the identification of individuals at high risk, facilitate the development of targeted treatments, and monitor treatment response. The following steps include validating these findings in larger cohorts and conducting clinical trials to evaluate interventions based on these variants. These advances could revolutionize the prediction and treatment of CRC, improving clinical outcomes and patient quality of life.

## 5. Conclusions

In conclusion, the analysis reveals a significant association between the genetic variants *RASSF1* (rs2073498), *SERPINE1* (rs1799889), and *EFNA1* (rs12904) and colorectal cancer, highlighting their potential as valuable genetic markers.

The in silico analyses provided critical insights into the possible functional impacts of these variants, indicating their involvement in gene regulation, protein functionality, and interactions with microRNAs.

Further investigations are necessary to validate these associations and explore therapeutic interventions targeting these variants, ultimately contributing to enhanced strategies for managing and treating CRC.

## Figures and Tables

**Table 1:** Demographic and clinical characteristics of colorectal cancer patients and control subjects.

Characteristics	CRC Group	Control Group	*p Value*
	*n* = 349 (100%)	*n* = 282 (100%)	
**Mean Age (Years SD)**	58.34 (±12.32) 20–92	59.76 (±13.28) 20–92	0.165
**Age (Years)**			
<50	68 (19.48)	25 (8.86)	**0.001**
>50	281 (80.52)	257 (91.14)	
**Sex**			
Male	202 (57.88)	141 (50)	0.048
Female	147 (42.12)	141 (50)	
**Tobacco Consumption**			
Yes	118 (33.81)	43 (15.25)	**0.001**
No	231 (66.19)	239 (84.75)	
**Alcohol Consumption**			
Yes	109 (31.23)	34 (12.06)	**0.001**
No	240 (68.77)	248 (87.94)	
**TNM Stage**			
I	7 (2.01)		
II	109 (31.23)		
III	130 (37.25)		
IV	103 (29.51)		
**Tumor Localization**			
Colon	74 (21.20)		
Recto	275 (78.80)		
**Metastasis Site**			
Liver	36 (34.95)		
Lung	11 (10.68)		
Liver and Lung	15 (14.56)		
Peritoneum	5 (4.85)		
Others	9 (8.74)		
Not available	27 (26.21)		
**Treatment Response**			
Complete Response	161 (46.13)		
Partial Response	85 (25.21)		
No Response	103 (28.65)		

*p*-values were calculated using the Chi-square test. The bold text highlights statistically significant results. Adjusted by the Bonferroni test (0.0125).

**Table 2:** Genotype and allele frequencies of *RASSF1*, *SERPINE1*, *EFNA1*, and *RAD51* variants in colorectal cancer patients and the control group.

Genotype	CRC *n* = 349 (100%)	Control *n* = 282 (100%)	OR (C.I. 95%)	*p*
***RASSF1* rs2073498 (Ala 133 ser)**				
Ala/Ala	126 (36.10%)	176 (62.41%)	1.00 (Reference)	**-**
Ala/Ser	**185 (53.01%)**	**100 (35.46%)**	**2.58 (1.85–3.60**)	**0.001**
Ser/Ser	**38 (10.89%)**	**6 (2.13%)**	**8.84 (3.62–21.56)**	**0.001**
Ala/Ser + Ser/Ser vs. Ala/Ala	**223 (63.90%)**	**106 (37.59%)**	**2.93 (2.12–4.06**)	**0.001**
**Allele**				
Ala	437 (62.61%)	452 (80.14%)	1.00 (Reference)	-
Ser	**261 (37.39%)**	**112 (19.86%)**	**2.41 (1.86–3.11)**	**0.001**
***SERPINE1* rs1799889**				
5G/5G	80 (22.92%)	97 (34.40%)	1.00 (Reference)	-
5G/4G	**213 (61.03%)**	**145 (51.42%)**	**1.78 (1.23–2.56)**	**0.002**
4G/4G	56 (22.92%)	40 (34.40%)	1.69 (1.02–2.80)	0.051
5G/4G + 4G/4G vs. 5G/5G	**269 (77.08%)**	**185 (65.60%)**	**1.76 (1.24–2.50)**	**0.001**
**Allele**				
5G	373 (53.44%)	339 (60.11%)	1.00 (Reference)	-
4G	325 (46.56%)	225 (39.89%)	1.31 (1.04–1.64)	0.020
***EFNA1* rs12904**				
A/A	75 (21.49%)	89 (31.56%)	1.00 (Reference)	-
A/G	161 (46.13%)	133 (47.16%)	1.43 (0.97–2.10)	0.079
G/G	**113 (32.38%)**	**60 (21.28%)**	**2.23 (1.44–3.46)**	**0.005**
A/G + G/G vs. A/A	**274 (78.51%)**	**193 (68.44%)**	**1.68 (1.17–2.41)**	**0.005**
**Allele**				
A	311 (44.56%)	311 (55.14%)	1.00 (Reference)	-
G	**387 (55.44%)**	**253 (44.86%)**	**1.52 (1.22–1.91)**	**0.001**
***RAD51* rs1801320**				
G/G	93 (26.65%)	84 (29.79%)	1.00 (Reference)	-
G/C	174 (49.85%)	150 (53.19%)	1.04 (0.72–1.51)	0.876
C/C	82 (23.50%)	48 (17.02%)	1.54 (0.97–2.45)	0.084
G/C + C/C vs. G/G	256 (73.35%)	198 (70.21%)	1.16 (0.82–1.65)	0.433
**Allele**				
G	360 (51.60%)	318 (56.40%)	1.00 (Reference)	-
C	338 (48.40%)	246 (43.60%)	1.21 (0.97–1.51)	0.099

Bold numbers represent statistically significant values. Adjusted by the Bonferroni test (0.0125).

**Table 3:** Association of the *RASSF1* rs2073498 (Ala 133 Ser) variant with demographic and clinical characteristics.

*RASSF1* rs2073498 (Ala 133 Ser)						
	CRC/Control			OR (95% IC); *p* Value		
Characteristic	Ala/Ala	Ala/Ser	Ser/Ser	Ala/Ser vs. Ala/Ala	Ser/Ser vs. Ala/Ala	Ala/Ser + Ser/Ser vs. Ala/Ala
**Age (Years)**						
<50	25/12	40/12	3/1	1.60 (0.62–4.10); 0.460	1.44 (0.13–15.33); 1.000	1.58 (0.62–4.01); 0.457
>50	101/164	145/88	35/5	**2.67 (1.86–3.84); 0.001**	**11.36 (4.31–29.96); 0.001**	**3.14 (2.20–4.47); 0.001**
**Sex**						
Male	64/83	114/57	24/1	**2.59 (1.64–4.07); 0.001**	**31.12 (4.10–236.23); 0.001**	**3.08 (1.97–4.82); 0.001**
Female	62/93	71/43	14/5	**2.47 (1.50–4.07); 0.001**	4.20 (1.43–12.25); 0.010	**2.65 (1.64–4.28); 0.001**
**Tobacco Consumption**						
Yes	38/33	68/9	12/1	**6.56 (2.84–15.15); 0.001**	10.42 (1.28–84.47); 0.020	**6.94 (3.10–15.55); 0.001**
**Alcohol Consumption**						
Yes	37/17	58/16	14/1	1.66 (0.75–3.69); 0.291	6.43 (0.78–52.97); 0.108	1.94 (0.89–4.24); 0.138
**TNM Stage**						
I + II	43/176	63/100	10/6	**2.57 (1.62–4.08); 0.001**	**6.82 (2.35–19.80); 0.001**	**2.81 (1.80–4.40); 0.001**
III + IV	83/176	122/100	28/6	**2.58 (1.78–3.75); 0.001**	**9.89 (3.94–24.81); 0.001**	**3.00 (2.09–4.30); 0.001**
I + II + III	88/176	132/100	26/6	**2.64 (1.83–3.80); 0.001**	**8.66 (3.44–21.83); 0.001**	**2.98 (2.09–4.25); 0.001**
IV	38/176	53/100	12/6	**2.45 (1.51–3.98); 0.001**	**9.26 (3.27–26.22); 0.001**	**2.84 (1.78–4.53); 0.001**
**Tumor Location**						
Colon	25/176	41/100	8/6	**2.88 (1.65–5.02); 0.001**	**9.38 (3.00–29.30); 0.001**	**3.25 (1.89–5.57); 0.001**
Rectum	101/176	144/100	30/6	**2.50 (1.76–3.57); 0.001**	**8.71 (3.50–21.64); 0.001**	**2.86 (2.02–4.03); 0.001**

The bold text highlights statistically significant results. Adjusted by the Bonferroni test (0.0125).

**Table 4:** Association of *SERPINE1* rs1799889 variant with demographic and clinical characteristics.

*SERPINE1* rs1799889						
	CRC/Control			OR (95% IC); *p* Value		
Characteristics	5G/5G	5G/4G	4G/4G	5G/4G vs. 5G/5G	4G/4G vs. 5G/5G	5G/4G + 4G/4G vs. 5G/5G
**Age (Years)**						
<50	10/6	43/14	15/5	1.84 (0.56–5.98); 0.478	1.80 (0.43–7.53); 0.656	1.83 (0.58–5.71); 0.457
>50	70/91	170/131	41/35	1.68 (1.14–2.48); 0.010	1.52 (0.88–2.63); 0.171	1.65 (1.13–2.39); 0.010
**Sex**						
Male	43/54	125/67	34/20	**2.34 (1.42–3.85); 0.001**	2.13 (1.07–4.22); 0.042	**2.29 (1.42–3.70); 0.001**
Female	37/43	88/78	22/20	1.31 (0.76–2.23); 0.391	1.27 (0.60–2.70); 0.650	1.30 (0.77–2.18); 0.380
**Tobacco Consumption**						
Yes	21/14	77/22	20/7	2.33 (1.02–5.32); 0.069	1.90 (0.63–5.69); 0.373	2.22 (1.00–4.92); 0.072
**Alcohol Consumption**						
Yes	22/17	70/13	17/4	**4.16 (1.74–9.89); 0.001**	3.28 (0.93–11.57); 0.105	**3.95 (1.74–8.96); 0.001**
**TNM Stage**						
I + II	25/97	73/145	18/40	**1.95 (1.15–3.29); 0.015**	1.74 (0.85–3.54); 0.172	1.90 (1.15–3.16); 0.016
III + IV	55/97	140/145	38/40	**1.70 (1.13–2.55); 0.012**	1.67 (0.96–2.91); 0.090	**1.69 (1.14–2.50); 0.010**
I + II + III	59/97	144/145	43/40	1.63 (1.09–2.42); 0.020	1.76 (1.03–3.02); 0.051	**1.66 (1.13–2.43); 0.011**
IV	21/97	69/145	13/40	**2.19 (1.26–3.81); 0.006**	1.50 (0.68–3.28); 0.416	**2.04 (1.19–3.50); 0.011**
**Tumor Location**						
Colon	22/97	39/145	13/40	1.18 (0.66–2.12); 0.669	1.43 (0.65–3.12); 0.481	1.23 (0.71–2.16); 0.535
Rectum	58/97	174/145	43/40	**2.00 (1.35–2.97); 0.001**	1.79 (1.04–3.08); 0.045	**1.96 (1.34–2.86); 0.001**

The bold text highlights statistically significant results. Adjusted by the Bonferroni test (0.0125).

**Table 5:** Association of the *EFNA1* rs12904 variant with demographic and clinical characteristics.

***EFNA1* rs12904 A>G**						
	**CRC/Control**			**OR (95% IC); *p* Value**		
**Characteristics**	**A/A**	**A/G**	**G/G**	**A/G vs. A/A**	**G/G vs. A/A**	**A/G + G/G vs. A/A**
**Age (Years)**						
<50	10/9	36/11	22/5	2.94 (0.95–9.07); 0.104	3.96 (1.05–14.88); 0.077	3.26 (1.13–9.38); 0.049
>50	65/80	125/120	91/55	1.28 (0.84–1.93); 0.281	**2.03 (1.27–3.25); 0.004**	1.51 (1.03–2.22); 0.040
**Sex**						
Male	49/47	88/62	65/32	1.36 (0.81–2.27); 0.297	1.94 (1.08–3.48); 0.034	1.56 (0.97–2.51); 0.085
Female	26/42	73/71	48/28	1.66 (0.92–2.99); 0.121	**2.76 (1.40–5.44); 0.004**	**2.22 (1.26–3.86); 0.007**
**Tobacco Consumption**						
Yes	25/15	59/21	34/7	1.68 (0.74–3.79); 0.290	2.91 (1.03–8.20); 0.069	1.99 (0.92–4.29); 0.115
**Alcohol Consumption**						
Yes	17/11	49/13	43/10	2.43 (0.92–6.46); 0.118	2.78 (0.99–7.74); 0.084	2.58 (1.06–6.27); 0.057
**TNM Stage**						
I + II	28/89	57/133	31/60	1.36 (0.80–2.30); 0.306	1.64 (0.89–3.01); 0.146	1.44 (0.88–2.37); 0.175
III + IV	47/89	104/133	82/60	1.48 (0.95–2.29); 0.097	**2.58 (1.59–4.20); 0.001**	**1.82 (1.21–2.74); 0.004**
I + II + III	56/89	116/133	74/60	1.36 (0.91–2.10); 0.152	**1.96 (1.21–3.15); 0.007**	1.56 (1.05–2.31); 0.030
IV	19/89	45/133	39/60	1.58 (0.87–2.88); 0.171	**3.04 (1.60–5.76); 0.001**	2.03 (1.16–3.56); 0.016
**Tumor Location**						
Colon	17/89	36/133	21/60	1.41 (0.75–2.67); 0.357	1.83 (0.89–3.75); 0.138	1.54 (0.85–2.80); 0.195
Rectum	58/89	125/133	92/60	1.44 (0.95–2.17); 0.100	**2.35 (1.47–3.74); 0.001**	**1.72 (1.17–2.53); 0.006**

The bold text highlights statistically significant results. Adjusted by the Bonferroni test (0.0125).

**Table 6:** Logistic regression analysis for CRC patients and the control group.

Independent Characteristics	**B ^1^**	**s.e ^2^**	**Wald ^3^**	**d.f ^4^**	***p* Value**	**OR (95% IC)**
**Age (Years) > 50 vs. <50**	0.696	0.269	6.697	1	**0.010**	2.00 (1.18–3.39)
**Sex Male vs. Female**	0.114	0.179	0.407	1	0.523	1.12 (0.79–1.59)
**Smoking Status Yes vs. No**	0.757	0.224	11.136	1	**0.001**	2.13 (1.37–3.30)
**Drinking Status Yes vs. No**	0.812	0.241	11.356	1	**0.001**	2.25 (1.40–3.61)
***RASSF1* Ala/Ser + Ser/Ser**	1.028	0.176	34.171	1	**0.001**	2.79 (1.98–3.94)
***SERPINE1* rs1799889** **5G/4G + 4G/4G**	0.504	0.194	6.706	1	**0.010**	1.65 (1.13–2.42)
***EFNA1* rs1732 AG + GG**	0.472	0.199	5.645	1	0.018	1.60 (1.08–2.36)
***RAD51* rs1801320 GC + CC**	0.124	0.195	0.405	1	0.525	1.132 (0.77–1.66)
**Constant**	−2.905	0.363	63.976	1	**0.001**	
**Model**	X^2^ = 107.990 d.f. = 8 *p* = **0.001**					

^1^ regression coefficient. ^2^ standard error. ^3^ Wald test. ^4^ degrees of freedom. The bold text highlights statistically significant results. Adjusted by the Bonferroni test (0.0125).

## Data Availability

The original contributions presented in the study are included in the article/Supplementary Materials, further inquiries can be directed to the corresponding author.

## Supplementary Materials

The following supporting information can be downloaded at https://www.mdpi.com/article/10.3390/genes16020223/s1, Figure S1: Expression Profiles of *RASSF1*, *SERPINE1* and *EFNA1*; in COAD and READ Patients and Controls; Figure S2: Gene Expression Profiles of *RASSF1*, *SERPINE1* and *EFNA1*; Across Different Tumor Stages; Figure S3: Gene expression profiles of *RASSF1*, *SERPINE1*, and *EFNA1*, across different tumor stages; Table S1: In silico analysis of *RASSF1*, *SERPINE1*, *EFNA1* and variants using CADD tool; Table S2: In silico predicted miRNAs associated with the *EFNA1* rs12904 variant in the 3′UTR Region by PolymiRTS bioinformatic tool.
