# Supplementary material for: Genetic Variants in RASSF1 (rs2073498), SERPINE1 (rs1799889), and EFNA1 (rs12904) Are Associated with Susceptibility in Mexican Patients with Colorectal Cancer: Clinical Associations and Their Analysis In Silico
Source: Genes (Basel). 2025 Feb 15;16(2):223. doi: 10.3390/genes16020223 (PMC11855561; doi:10.3390/genes16020223)
Supplement: Supplementary file 1 [file genes-16-00223-s001.zip › Supplementary Figures/Supplementary figure legends.pdf]

# Supplementary figure legends

**Figure S1.** Expression Profiles of *RASSF1, SERPINE1* and *EFNA1*; in COAD and READ Patients and Controls
The figure presents box-and-whisker plots comparing the expression profiles of four genes *(RASSF1, SERPINE1, EFNA1,)* in patient and control samples. The GEPIA analysis focuses on mean expression levels in Colorectal adenocarcinoma (COAD) and Rectal adenocarcinoma (READ) compared to their respective controls. Each plot is divided into two sections: one for COAD and one for READ.
**Supplementary Figure S1. (a) *RASSF1*:** Slight under-expression in both COAD (num(T)=275; num(N)=349) and READ patients compared to controls, although this reduction was not statistically significant.
**Supplementary Figure S1.** (**b) *SERPINE1*:** Slight overexpression in COAD and READ patients compared to controls; however, this difference did not achieve statistical significance.
**Supplementary Figure S1. (c) *EFNA1***: Slight overexpression was observed in both COAD and READ patients compared to controls, though not statistically significant.

**Figure S2.** Gene Expression Profiles of *RASSF1, SERPINE1* and *EFNA1;* Across Different Tumor Stages.
The figure presents violin plots comparing the expression profiles of four genes (*RASSF1, SERPINE1* and *EFNA1*) across different tumor stages (Stage I, II, III, and IV).
**Supplementary Figure S2 (a) *RASSF1***: The plot shows the expression levels of *RASSF1* with an F value of 2.72 and a p-value of 0.0447, indicating statistically significant differences among the stages.
**Supplementary Figure S2 (b) *SERPINE1***: This plot displays the expression levels of *SERPINE1*. Its F value is 5.28, and its p-value is 0.00142, indicating statistically significant overexpression across the stages.
**Supplementary Figure S2 (c) *EFNA1***: The expression levels of *EFNA1* are shown, with an F value of 3.67 and a p-value of 0.0126, highlighting significant differences across tumor stages.

**Figure S3.** Gene expression profiles of *RASSF1, SERPINE1,* and *EFNA1,* across different tumor stages. This eQTL assay investigates the association between genetic variants (*RASSF1* rs2073498 and *EFNA1* rs12904) and gene expression levels across various tissues (Whole Blood, Colon—transverse, and Colon—sigmoid). Each panel presents a violin plot depicting the normalized expression levels of the respective genes across different genotypes.
