# Supplementary material for: Genetic Variants in RASSF1 (rs2073498), SERPINE1 (rs1799889), and EFNA1 (rs12904) Are Associated with Susceptibility in Mexican Patients with Colorectal Cancer: Clinical Associations and Their Analysis In Silico
Source: Genes (Basel). 2025 Feb 15;16(2):223. doi: 10.3390/genes16020223 (PMC11855561; doi:10.3390/genes16020223)
Supplement: Supplementary file 1 [file genes-16-00223-s001.zip › Supplementary Tables/Table S1.pdf]

**Supplementary Table S1**: In-silico analysis of *RASSF1*, *SERPINE1*, *EFNA1* and variants using CADD tool

| Gene | Chromosome | Cons detail | Min Dist TSE | Target Scan | Mir SVR-Score | Mir SVR-E | Mir SVRA ln | Ensemble Regulatory Feature | SIFT cat | SIFT val | Poly Phen Cat | Poly Phen Val | Esm Score Missense | Gerp RS | GerpR Spval | Raw Score | PHRED |
|---|---|---|---|---|---|---|---|---|---|---|---|---|---|---|---|---|---|
| *RASSF1* | 3 | missense | N/A | N/A | N/A | N/A | N/A | N/A | Tolerated | 0.26 | Benign | 0.013 | -5.56 | N/A | N/A | 2.85524 | 20.6 |
| *SERPINE1* | 7 | upstream | 675 | N/A | N/A | N/A | N/A | Promoter | N/A | N/A | N/A | N/A | N/A | 346.19 | 8.2E-20 | 1.969,031 | 16.61 |
| *EFNA1* | 1 | 3_UTR | 87 | 9 | -1.088 | -13.8 | 150 | Promoter | N/A | N/A | N/A | N/A | N/A | N/A | N/A | 2.330,925 | 18.26 |

The table provides a comprehensive summary of the results derived from the CADD analysis, including CADD scores, the types of identified mutations, and their corresponding functional impacts on the genes under examination. The c-phred values denote the likelihood that a given variant is functional, with higher values indicating an increased probability of functional significance.
