# Supplementary material for: Genetic Variants in RASSF1 (rs2073498), SERPINE1 (rs1799889), and EFNA1 (rs12904) Are Associated with Susceptibility in Mexican Patients with Colorectal Cancer: Clinical Associations and Their Analysis In Silico
Source: Genes (Basel). 2025 Feb 15;16(2):223. doi: 10.3390/genes16020223 (PMC11855561; doi:10.3390/genes16020223)
Supplement: Supplementary file 1 [file genes-16-00223-s001.zip › Supplementary Tables/Table S2.pdf]

Supplementary Table S2. In-silico predicted miRNAs associated with the *EFNA1* rs12904 variant in the 3'UTR Region by PolymiRTS bioinformatic tool.

Location	dbSNP ID	Variant type	miR ID	Conservation	Function Class	Experimental Support	context+ score change	Target Gene ID**
155106697	rs12904	SNP	miR-200b-3p	10	D	N	-0.17	MAP3K3, AMDHD2, ETS1, PHC2, RPGRIP1L, CDH11, SYBU, APBB2, CEP78, ZNF518A
			miR-200c-3p	10	D	N	-0.17	*CLUAP1, RAB37, LRRC7, LIMCH1, CMC1, PPIP5K2, ZNF383, FLRT2, USP28, CEP83*
			miR-374c-5p	9	D	N	0.028	*SANBR, CLUAP1, CDH11, OXNAD1, TMEM268, DYRK1A, ZNF148, CFAP92, KIDINS220, CAMTA1*
			miR-429	10	D	N	-0.17	*MTA3, ZNF423, TPP2, EIF4B, NTNG1, YTHDC1, SIRPA, GAPVD1, CMC1, TRIM52*
			miR-4750-3p	9	D	N	-0.246	*LARP4, NAGK, FSD1, CD300A, RAB37, CAP1, AKAIN1, GRHL2, IQSEC1, CUL4B*
			miR-655-3p	9	D	N	0.028	*HELZ, FSD1L, DAZL, ATP11C, ANKRD54, TENT4B, ATF7, ATF7, DACH1, INTS4*
			miR-8084	10	D	N	-0.002	*PARP8, TMX3, ZBTB41, OR2C3, KANTR, RRM2B, NSUN4, CLCN4, ZNF143, SLC39A10*
			miR-888-3p	9	D	N	-0.14	*PARP8, TMX3, ZBTB41, OR2C3, KANTR, RRM2B, NSUN4, CLCN4, ZNF143, SLC39A10*

D: Disruptive
N: No Experimental support
