# Supplementary figures and images for: Genetic Variants in RASSF1 (rs2073498), SERPINE1 (rs1799889), and EFNA1 (rs12904) Are Associated with Susceptibility in Mexican Patients with Colorectal Cancer: Clinical Associations and Their Analysis In Silico
Source: Genes (Basel). 2025 Feb 15;16(2):223. doi: 10.3390/genes16020223 (PMC11855561; doi:10.3390/genes16020223)

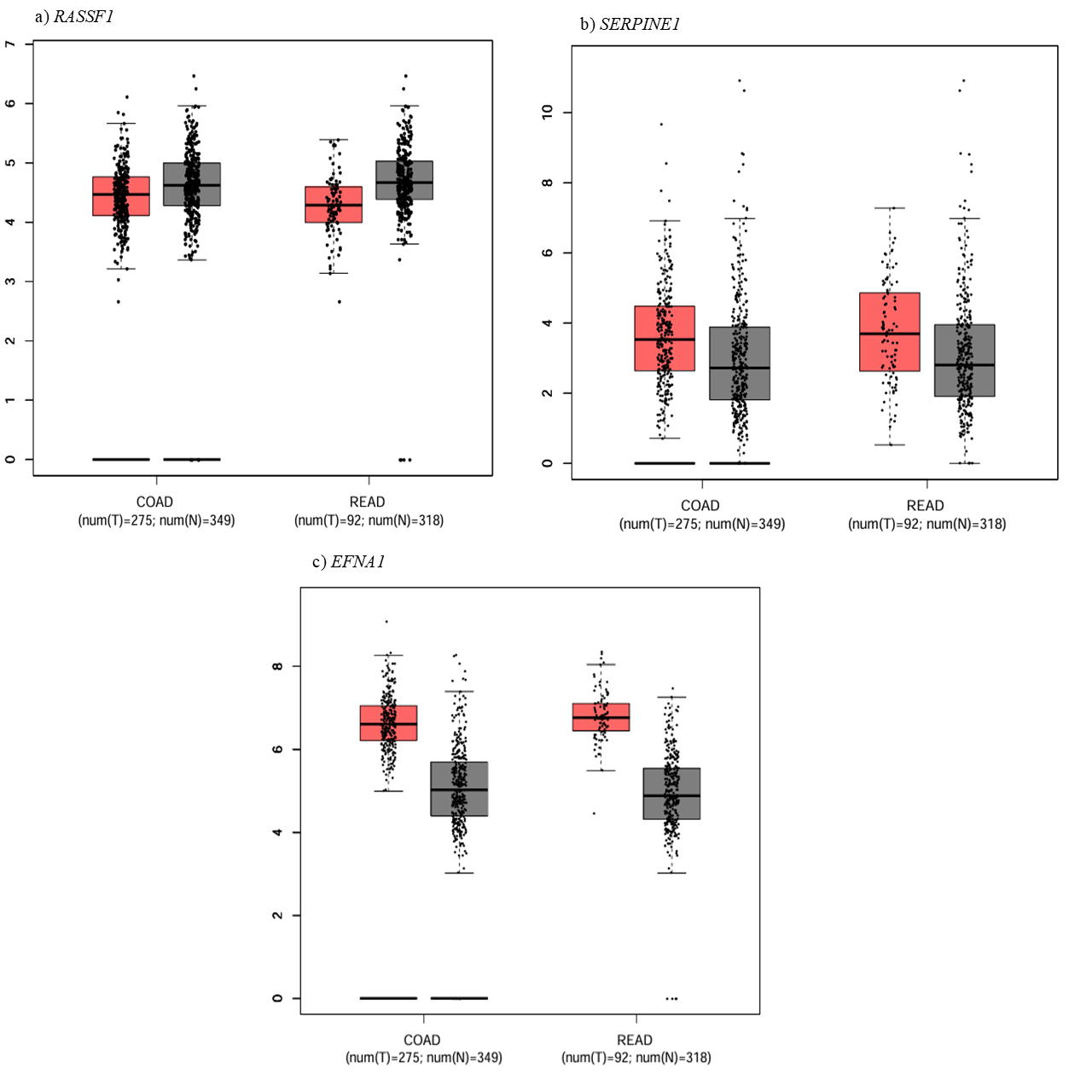

Supplement: Supplementary file 1 [file genes-16-00223-s001.zip › Supplementary Figures/Figure S1.jpeg]

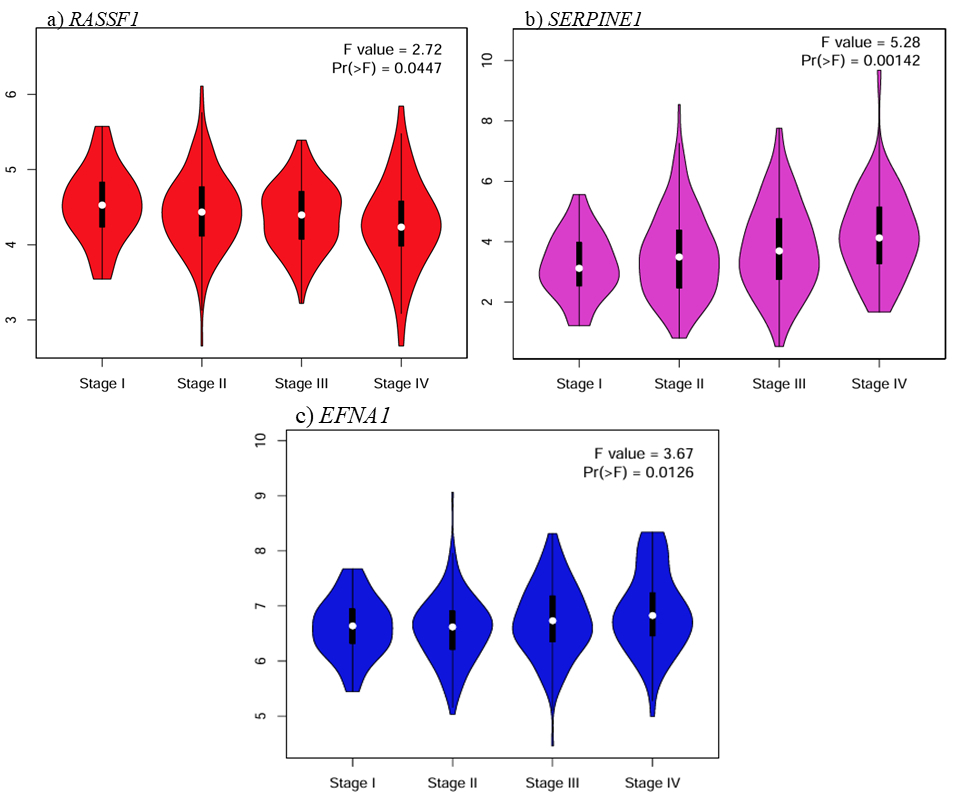

Supplement: Supplementary file 1 [file genes-16-00223-s001.zip › Supplementary Figures/Figure S2.jpeg]

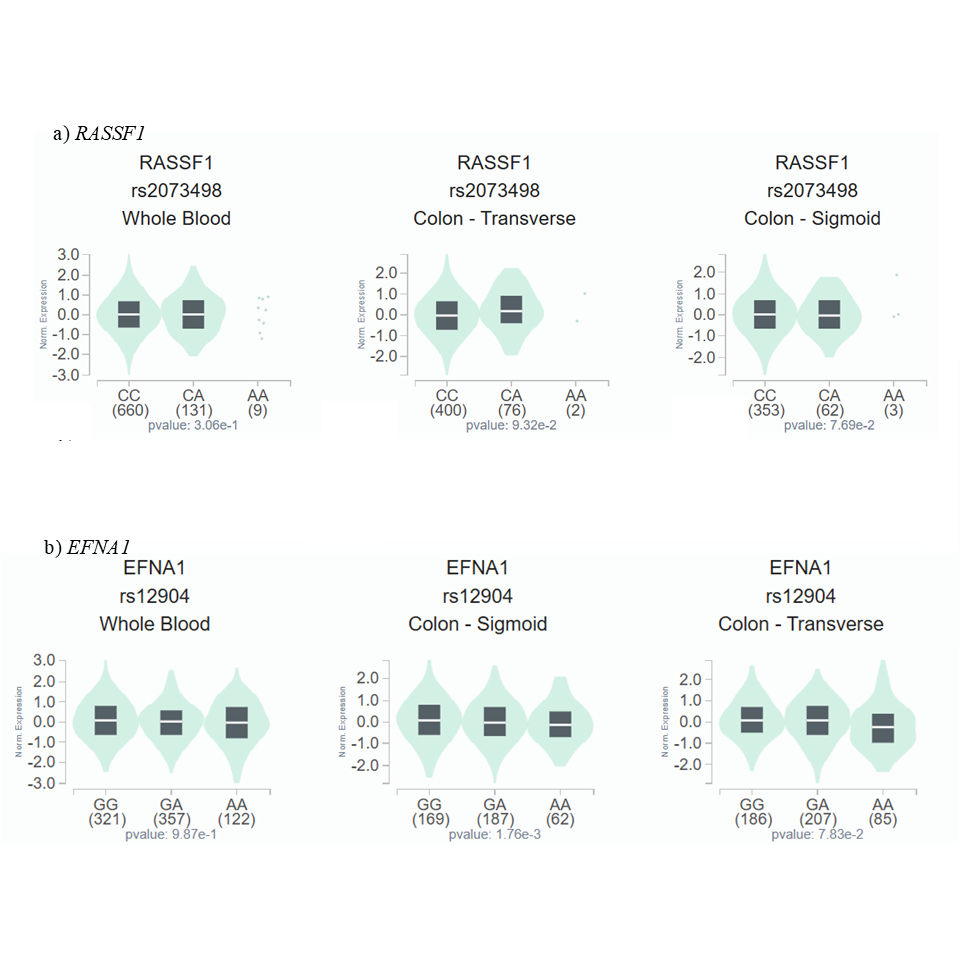

Supplement: Supplementary file 1 [file genes-16-00223-s001.zip › Supplementary Figures/Figure S3.jpeg]
